# Solitary Plasmacytoma of the Calcaneum: A Case Report

**DOI:** 10.31729/jnma.4566

**Published:** 2019-10-31

**Authors:** Saugat Poudyal, Shailendra Shrestha

**Affiliations:** 1 Department of Clinical Oncology, National Academy of Medical Sciences, Mahaboudha, Kathmandu, Nepal; 2 Department of Orthopaedics, Nepal Armed Police Force Hospital, Balambu, Kathmandu, Nepal

**Keywords:** *age groups*, *calcaneus*, *plasmacytoma*, *trauma*

## Abstract

Solitary Plasmacytoma is characterized by a mass of neoplastic monoclonal plasma cells in either bone or soft tissue without evidence of systemic disease attributing to myeloma. Solitary bony plasmacytoma commonly presents in the axial skeleton. The occurrence of solitary bony plasmacytoma in young individuals is exceedingly rare but has been reported sporadically. We report a case of Solitary bony plasmacytoma involving the calcaneum (appendicular skeleton) in a 26-year-old male with a prior history of trauma at the same site. To our knowledge, this is one of the few cases of solitary bony plasmacytoma in the calcaneum being reported. Our patient presents with some unusual features like the unusual site of the lesion (calcaneum), presentation at a young age and the disease being symptomatic following trauma.

## INTRODUCTION

Solitary plasmacytoma of bone (SPB) is a localized tumor in the bone composed of a single clone of plasma cells in the absence of features of multiple myeloma such as anemia, hypercalcemia, renal insufficiency, or multiple lytic bone lesions.^1^ It constitutes about 5% of all plasma cell disorders. The median age at diagnosis is 55 to 65 years and they present with skeletal pain or pathological fracture.^2^ SPB occurs more commonly in bones of the axial skeleton such as vertebra and skull.^2^ Involvement of the appendicular skeleton is less frequent and calcaneum is a very rare site for SBP.

To the best of our knowledge, this is one of few cases of SBP with some unusual clinical features, the involvement of appendicular skeleton-like calcaneum, an occurrence at a young age and finally the disease being symptomatic following trauma.^3,4^

## CASE REPORT

A twenty-six years gentleman was evaluated for complaints of constant pain in the left hind foot following a trauma one year back. Initially, he was prescribed some analgesics by the local practitioner with some temporary relief. A year after, there was increasing pain with associated swelling in the left calcaneal region for which he sought a consultation. An MRI of the left ankle showed an abnormal high Short T1 Inversion Recovery (STIR) changes involving the calcaneum in keeping with marrow edema. There was minimal edema in the surrounding soft tissue with no associated mass or a periosteal reaction or collection and was reported as an inflammatory change. With the diagnosis of Atypical rheumatoid arthritis, he was prescribed a course of steroids and analgesics.

The symptoms still unremitting, and the patient limping with pain, he came to us for further consultation. On examination, there was local swelling with mild to moderate tenderness in the calcaneal region. There was no apparent discoloration of the swelling site and the movement of the left ankle joint was normal. A biopsy was done from the left calcaneum which was suggestive of Plasmacytoma. A whole-body MRI scan was negative for other skeletal involvement. The patient’s blood counts were as follows, hemoglobin=15g%, total leukocyte count= 9800 cells/mm^3^ and platelet count=250*10^6^ /L. Serum calcium was 10.7 mg/dL (corrected calcium 10.1 mg/dl). Urea was 15mg/dl and creatinine was 1.1 mg/dl. His peripheral smear, bone marrow aspirate, and trephine were within the normal limits. Urine Bence-Jones protein was negative. An immunoglobulin assay was done. IgG was 17.4 g/dl (Range 7-16 g/dl), IgA was 2.10 g/dl (Range 0.7-4 g/dl), IgM was 2.08 g/dl (Range 0.4-2.3 g/dl). Free kappa and free lambda light chain ratios were 1.25 (normal range= 0.31-1.56). M-spike was not detected. Beta 2 microglobulin (Nephelometry method) was 2190 ng/ml (Range 700-1800 ng/ml). Human immunodeficiency virus, hepatitis B surface antigen, and hepatitis C virus (HCV) were tested for and were negative. There was no history of any malignancy in the family. Based on the above features of exclusion of multiple myeloma and biopsy finding of plasmacytoma, no other differentials were considered and a diagnosis of SPB was made as per diagnostic criteria for solitary plasmacytoma, recommended by the International Myeloma Working Group.^1^ The patient was taken up for local radiotherapy with 45 Gray in 25 fractions. During the course of radiation, he gradually improved symptomatically. His pain was decreased and was able to visit us after a month of completion of treatment, without any supports. A response evaluation MRI scan is planned after 3 to 6 months and he will be monitored 6 monthly with clinical assessment, a complete blood count, serum chemistry (calcium, creatinine), serum and urine testing for M-protein, to detect progression to multiple myeloma.

## DISCUSSION

SBP commonly occurs in the spine, ribs, pelvis, skull, sternum, and proximal long bones of the extremities. There have been reported cases of SBP at unusual sites like the bones of hand and foot. The cause of this unusual presentation is largely unknown. Such an uncommon site, like bones of the foot, are rare for even primary tumors of the bone. The differentials of primary tumors include osteogenic sarcoma, chondrosarcoma, fibrosarcoma, liposarcoma, and round cell tumors. There also have been reported cases of metastatic lesion of the foot from colon, renal, breast and lung primaries, which too are rare.^5^ A common diagnosis for patients presenting in the clinic with calcaneal swelling and associated tenderness would be Osteomyelitis. They, however, present with signs of local inflammation and other constitutional symptoms like pyrexia and the presence of puncture wounds. Plantar fasciitis with calcaneal enthesopathy or a seronegative spondyloarthropathy can produce calcaneal bone marrow edema, as in our case with MRI finding of marrow edema.^6^ The radiological diagnostic problem in our case was complicated by the fact that the patient had a history of trauma leading the radiologist to suspect the lesion being trauma-induced.

The median age for SBP is 55 years. Its occurrence in young individuals is exceedingly rare. To our knowledge, a total of 14 cases have been reported in young adults. The age of all of these cases including our’s is less than 30 years.^7-9^ The majority of these cases had involvement of the spine (7 cases), 3 with the tibial lesion, 2 with rib involvement, 1 involving the vertex of the skull and 1 involving the metacarpal. The involvement of calcaneum with SBP at a younger age has rarely been reported. Tibial involvement and its correlation in the young age group has been suggested but lacks proper proof.^7^ The disease being apparent at a young age may suggest an indolent nature of multiple myeloma since most of the cases of SBP progress to multiple myeloma.^1^ The presentation as such, at a young age following a history of trauma could merely be a continuum of the spectrum of disease which became apparent early due to trauma.

The etiology of SPB remains uncertain, but several hypotheses have been proposed implicating the role of radiation, chemical exposure, viruses, and genetic factors.^1^ Cytogenetic studies revealed loss in chromosome 13, 1p, 14q and gain in 19p, 9q, 1q, and interleukin-6 are considered as principal growth factors in pathogenesis. A study has also questioned the association of preceding trauma in young patients and the manifestation of SBP.^8^

The stimulation of mature naïve B cells is the hallmark of normal plasma cell development, it’s proliferation, heavy chain class switching and differentiation into memory B cells or plasma cells.^10^ Trauma can cause an enhanced release of cytokine resulting in increased proliferation of plasma cells and stromal cells in the bone. Concomitant expression of MYC and deregulated IL-6 led to a more rapid plasmacytoma occurrence in a mouse model. Given that IL-6 is a well known inflammatory cytokine that promotes B-cell proliferation occurring during bone marrow stimulation, the authors postulated that this creates a “feed-forward cytokine amplification loop” in which the tumor environment becomes ideal for plasma cell neoplasm progression.^11^ The trauma of any sort can trigger injured cells to secrete these inflammatory cytokines, which can sometimes lead to plasmacytoma development.

The exact significance of preceding trauma as a cause or a triggering stimulus for the development of solitary plasmacytoma is a debatable issue. If we consider, the nature of the disease with almost all cases of SBP progressing to Multiple myelomas, it can be argued that SBP is merely a part of the spectrum of Multiple myelomas and that the disease is apparent following a trauma suggests that trauma may act as a triggering stimulus in the pathogenesis of the disease. There are a considerable number of reported cases with a preceding history of trauma in patients with SBP.^11^

Although our patient has improved symptomatically, he will require frequent monitoring. Of interest, one of the larger published series of SBP with patients treated prior to 2001 (n= 206) indicated that the progression rate to multiple myeloma following radiotherapy is more rapid in the first 3 years (14% per year) than in the subsequent 7 years (3% to 4% per year), reaching a 10-year rate of 65%.^6^ Many differentials exist for a hindfoot pain as discussed above. SBP of the calcaneum is a very rare diagnosis and should be considered in cases of persistent pain in spite of aggressive management.

Consent: JNMA Case Report Consent Form was signed by the patient and the original article is attached with the patient’s chart.

## Conflict of Interest:

None.

## References

[1] Rajkumar SV, Dimopoulos MA, Palumbo A, Blade J, Merlini G, Mateos MV (2014). International Myeloma Working Group updated criteria for the diagnosis of multiple myeloma. Lancet Oncol..

[2] Dimopoulos MA, Moulopoulos A, Delasalle K, Alexanian R (1992). Solitary Plasmacytoma of Bone and Asymptomatic Multiple Myeloma. Hematol Oncol Clin North Am..

[3] Sprinkle RL, Santangelo L, DeUgarte R (1988). Solitary plasmacytoma of bone in the calcaneus. J Am Podiatr Med Asso..

[4] Shrimali R, Garg L, Setia V, Jain S (2002). Solitary plasmacytoma of the calcaneum SPC)- Radiograph and CT findings in SPC: a rare bone tumor. Indian J Radiol Imaging..

[5] Wu KK, Guise ER (1978). Metastatic tumors of the Foot. South Med J..

[6] Williams SK, Brage M (2004). Heel pain-plantar fasciitis and Achilles enthesopathy. Clin Sports Med..

[7] Rago A, Miraglia A, Mecarocci S, Pisanelli GC, Chiappetta C, Di Cristofano C (2010). Solitary plasmacytoma of tibia: a possible correlation with younger age. Leuk Res..

[8] Pasch W, Zhao X, Rezk SA (2012). Solitary plasmacytoma of the bone involving young individuals, is there a role for preceding trauma?. Int J Clin Exp Pathol..

[9] Jacob PM, Nair RA, Koshy SM, Kattoor J (2014). Solitary plasmacytoma of the metacarpal bone in an adolescent. Indian J Pathol Microbiol..

[10] Reza SA, Weiss LM (2007). Epstein-Barr virus-associated lymphoproliferative disorders. Human Pathology..

[11] Hussein MA, George R, Rybicki L, Karam MA (2003). Skeletal trauma preceding the development of plasma cell dyscrasia: Eight case reports and review of the literature. Med Oncol..

